# Surveillance of medically‐attended influenza in elderly patients from Romania—data from three consecutive influenza seasons (2015/16, 2016/17, and 2017/18)

**DOI:** 10.1111/irv.12752

**Published:** 2020-05-15

**Authors:** Daniela Pițigoi, Anca Streinu‐Cercel, Alina Elena Ivanciuc, Mihaela Lazãr, Carmen Maria Cherciu, Maria Elena Mihai, Maria Nițescu, Victoria Aramă, Maria Dorina Crăciun, Adrian Streinu‐Cercel, Oana Săndulescu

**Affiliations:** ^1^ Carol Davila University of Medicine and Pharmacy Bucharest Romania; ^2^ National Institute for Infectious Diseases “Prof. Dr. Matei Balș” Bucharest Romania; ^3^ “Cantacuzino” National Medico‐Military Institute for Research and Development Bucharest Romania; ^4^ Grigore Alexandrescu Clinical Children's Emergency Hospital Bucharest Romania

**Keywords:** case fatality, comorbidities, elderly, influenza, subtype

## Abstract

**Background:**

Influenza is an acute infection affecting all age groups; however, elderly patients are at an increased risk. We aim to describe the clinical characteristics and the circulation of influenza virus types in elderly patients admitted for severe acute respiratory infection (SARI) to a tertiary care hospital in Bucharest, Romania, part of the I‐MOVE+ hospital network.

**Methods:**

We conducted an active surveillance study at the National Institute for Infectious Diseases “Prof. Dr Matei Balș,” Bucharest, Romania, during three consecutive influenza seasons: 2015/16, 2016/17, and 2017/18. All patients aged 65 and older admitted to our hospital for SARI were tested for influenza by PCR.

**Results:**

A total of 349 eligible patients were tested during the study period, and 149 (42.7%) were confirmed with influenza. Most patients, 321 (92.5%) presented at least one underlying condition at the time of hospital admission, the most frequent being cardiovascular disease, 270 (78.3%). The main influenza viral subtype circulating in 2015/16 was A(H1N1)pdm09, followed by A(H3N2) in 2016/17 and B influenza in 2017/18. Case fatality was highest in the 2015/16 season (3.7%), 0% in 2016/17, and 1.0% in 2017/18. Vaccination coverage in elderly patients with SARI from our study population was 22 (6.3%) over the three seasons.

**Conclusions:**

Our study has highlighted a high burden of comorbidities in elderly patients presenting with SARI during winter season in Romania. The influenza vaccine coverage rate needs to be substantially increased in the elderly population, through targeted interventions.

## 1 BACKGROUND

Influenza is an acute infection affecting all age groups; however, elderly patients are at an increased risk, particularly due to a clustering of comorbidities which puts them at risk of severe influenza, complicated influenza, or influenza‐related decompensation of underlying conditions.^1^

On a global level, in 2017, influenza has been estimated to be responsible for 54 481 000 episodes of medically‐diagnosed lower respiratory tract infections leading to 9 459 000 hospitalizations, and specifically in Romania, with a population in 2017 of 19.6 million people, 174 000 episodes of lower respiratory tract infections with 65 000 hospitalizations, as calculated by the Global Burden of Disease Study 2017.^2^ Continued surveillance of influenza is warranted in each country, in order to better inform public health policies and to fill the existing information gaps, particularly for specific influenza risk groups.

In Romania, influenza surveillance is performed at national level by two surveillance systems, one for severe acute respiratory infections (SARI: testing one patient hospitalized with SARI per week from 20 hospitals throughout six counties, during the influenza surveillance season), and one for influenza‐like illness (ILI: testing all patients with ILI attending 192 sentinel general practitioners from 16 counties every Tuesday).^3^

The National Institute for Infectious Diseases “Prof. Dr Matei Balș,” a tertiary care hospital in Bucharest, Romania, was part of the I‐MOVE+ hospital network (http://www.i‐moveplus.eu/) from 2015 to 2018 and implemented a protocol for systematic screening for influenza in elderly patients admitted to the hospital for SARI.^4^

Here, we aim to describe the clinical features of the elderly patients admitted with SARI in our hospital and the circulation of influenza virus types in these patients during three consecutive influenza seasons in Bucharest, Romania, in order to characterize the epidemiology of influenza in this particular patient population, at high risk for influenza‐related morbidity.

## 2 METHODS

An active epidemiologic surveillance study was implemented at the National Institute for Infectious Diseases “Prof. Dr Matei Balș,” Bucharest, Romania, during three consecutive influenza seasons: 2015/16, 2016/17, and 2017/18. The study consisted of systematic daily screening of all consecutive admissions in patients aged 65 and older admitted to our hospital with an acute (onset <7 days) illness meeting the following SARI case definition: one or more general signs or symptoms, defined as: fever/feverishness, malaise, headache, myalgia, and altered clinical state (asthenia, anorexia, confusion, or weight loss) associated with one or more respiratory signs or symptoms defined as: cough, odynophagia, and dyspnea.^5^

Patients were excluded from the study if they met any of the following exclusion criteria: had a contraindication for influenza vaccine, had SARI onset ≥48 hours after hospital admission, were unwilling to participate or unable to communicate and give consent (either by the patient or by her/his legal representative), were institutionalized, had a respiratory specimen taken ≥8 days after SARI onset, and had tested positive for any influenza virus in the current season before the onset of symptoms leading to the current hospitalization. No information was collected on the number of exclusions overall and by each criterion, and therefore, this information is not available and will not be reported in Results.

For each consenting patient, a standardized medical questionnaire was filled out by study investigators through review of medical (hospital or general practitioner) records and patient/relative interview, collecting demographic variables, a complete medical history, influenza vaccination status in the respective season, and data related to the current SARI episode's onset, characteristics and outcome, as previously described.^6^ Two respiratory specimens (a nasal swab and a pharyngeal swab) were collected according to the methodology for national surveillance of influenza, ARI, and SARI,^3^ placed at 4°C immediately, and transported within 24 hours at 2‐8°C to the National Reference Laboratory (NRL) at the “Cantacuzino” National Medico‐Military Institute for Research and Development, Bucharest, Romania.

At the NRL, detection, typing, subtyping, and genetic lineages differentiation of influenza viruses were done using real‐time reverse transcription‐polymerase chain reaction (RT‐PCR). All samples were tested for the presence of influenza A and B viruses. For influenza A viruses, a second real‐time RT‐PCR analysis was performed for determination of A/H1 pdm09 or A/H3 subtype. The sequences and protocols for type A, B and subtype A/H3 primers and probes were obtained from Erasmus Medical Center Rotterdam. For detection of subtype A/H1pdm09, we used the protocols from CDC Atlanta in the season 2015/2016 and starting with the 2016/2017 season the WHO protocol (Molecular diagnosis influenza virus humans update 2014).^7^ For a proportion of influenza B viruses, a second real‐time RT‐PCR analysis (single nucleotide polymorphism—SNP) was performed for lineage determination: Yamagata or Victoria‐like lineage. MGB probes were designed for both B virus lineages that can be detected and discriminated simultaneously, as only one of the two probes will give a fluorescent signal.^8^ All real‐time RT‐PCR reactions were performed using commercial kits—SuperScript^®^ One‐Step qRT‐PCR System (Invitrogen).

For sequencing, influenza‐positive samples were passaged in adherent MDCK‐SIAT1 cells. Genetic characterization was done by sequencing the hemagglutinin (HA) coding region by Sanger sequencing using the BigDye Terminator v3.1 Cycle Sequencing kit (Applied Biosystems). The HA sequences were analyzed using the commercial software Sequencher® version 5.4.6 DNA sequence analysis software (Gene Codes Corporation).

Phenotypic testing of inhibition of neuraminidase (NA) activity for viral susceptibility to oseltamivir was performed with the fluorescence kit (NA‐Fluor™ Assay, MUNANA‐Methylumbelliferyl‐N‐acetylneuraminic acid substrate; Thermo Fisher Scientific).

Screening for influenza in our study was performed each season during the duration of the declared influenza season, based on the results of national surveillance, as follows: from week 53/2015 to week 20/2016 in the first influenza season, from week 48/2016 to week 18/2017 in the second season, and from week 50/2017 to week 17/2018 in the third season.

The study protocol was approved by the Ethics Committee of the “Cantacuzino” National Medico‐Military Institute for Research and Development—approvals number 46/03.09.2015, 108/07.09.2016, and 251/14.09.2017. Written informed consent was obtained from all subjects prior to inclusion in the study.

We report descriptive data as number and percentage for categorical variables, and as median and interquartile range (IQR) or range for non‐parametric continuous variables. Statistical associations were tested using the chi‐squared test for categorical variables and Mann–Whitney's *U* test for continuous non‐parametric variables. Two‐tailed *P* values <.05 were interpreted as statistically significant. IBM SPSS Statistics for Windows, version 20 (IBM Corp.) was used for the statistical analysis.

## 3 RESULTS

A total of 349 eligible patients were tested in this study, ranging between 53 and 191 by season included in the study. The baseline characteristics overall and by each of the three influenza seasons are presented in Table 1. The median (IQR) age was 74 (68, 80) years, and 43.6% of patients were men.

**TABLE 1:** Characteristics of patients included in the study in the three influenza seasons analyzed

Studied variable	2015/16 (n = 191)	2016/17 (n = 53)	2017/18 (n = 105)	Overall (n = 349)
Male gender	88 (46.1%)	26 (49.1%)	38 (36.2%)	152 (43.6%)
Age, median (IQR)	74 (67, 80)	77 (71, 85)	73 (69, 78.5)	74 (68, 80)
Smoking status				
Never smoked	138 (72.6%)	42 (79.2)	68 (64.9%)	248 (71.3%)
Former smoker	39 (20.5%)	9 (17.0%)	26 (24.8%)	74 (21.3%)
Current smoker	13 (6.8%)	2 (3.8%)	11 (10.5%)	26 (7.5%)
Vaccinated against influenza in the current seasonal	15 (7.9%, 95% CI: 4.5%‐12.7%)	2 (3.8%, 95% CI: 0.5%‐13.0%)	5 (4.8%, 95% CI: 1.6%‐10.8%)	22 (6.3%, 95% CI: 4.0%‐9.4%)
Comorbidities				
Patients with comorbidities^a^	177 (93.2%)	49 (92.5%)	95 (91.3%)	321 (92.5%)
Number of comorbidities, median (range)^a^	2 (0‐6)	2 (0‐4)	2 (0‐5)	2 (0‐6)
Frailty index, median (IQR)^a^	0.2 (0.1, 0.3)	0.2 (0.1, 0.3)	0.2 (0.1, 0.3)	0.2 (0.1, 0.3)
Cardiovascular disease^b^	149 (78.4%)	42 (79.2%)	79 (77.5%)	270 (78.3%)
Diabetes^b^	66 (34.7%)	15 (28.3%)	31 (30.4%)	112 (32.5%)
Obesity^c^	55 (28.9%)	10 (19.2%)	33 (31.4%)	98 (29.2%)
Chronic pulmonary disease^b^	48 (25.3%)	10 (18.9%)	20 (19.6%)	78 (22.6%)
Chronic liver disease^d^	6 (3.2%)	6 (11.3%)	9 (8.7%)	21 (6.1%)
Chronic kidney disease^e^	30 (15.9%)	7 (13.5%)	10 (9.7%)	47 (13.7%)
Hematologic cancer^a^	6 (3.2%)	2 (3.8%)	1 (1.0%)	9 (2.6%)
Non‐hematologic cancer^a^	18 (9.5%)	1 (1.9%)	12 (11.5%)	31 (8.9%)
Rheumatologic disease^f^	30 (15.9%)	7 (13.2%)	11 (11.0%)	48 (14.0%)
Immune suppression^a^	4 (2.1%)	0 (0.0%)	5 (4.8%)	9 (2.6%)
Laboratory results				
Laboratory‐confirmed influenza A^g^	66 (97.1%)	18 (90.0%)	9 (14.8%)	93 (62.4%)
Subtype A(H1N1)pdm09^h^	59 (89.4%)	0 (0.0%)	2 (22.2%)	61 (65.6%)
Subtype A(H3N2)^h^	7 (10.6%)	18 (100%)	7 (77.8%)	32 (34.4%)
Laboratory‐confirmed influenza B^g^	2 (2.9%)	2 (10.0%)	53 (86.9%)	57 (38.3%)^i^
Lineage B/Victoria^j^	N/A	2 (100%)	0 (0.0%)	2 (3.5%)
Lineage B/Yamagata^j^	N/A	0 (0.0%)	6 (11.3%)	6 (10.5%)
Lineage not determined^j^	2 (100%)	N/A	47 (88.7%)	49 (86.0%)
Antiviral treatment	118 (61.8%)	30 (56.6%)	81 (77.1%)	229 (65.6%)
Death during hospitalization	7 (3.7%, 95% CI: 1.5%‐7.4%)	0 (0.0%, 95% CI: 0.0%‐6.7%)	1 (1.0%, 95% CI: 0.0%‐5.2%)	8 (2.3%, 95% CI: 1.0%‐4.5%)

Note

All data is presented as number (percentage), unless otherwise specified. Frailty index was calculated by dividing the number of active comorbidities by the total number of comorbidities assessed for each patient.

Abbreviations: IQR, interquartile range; N/A, not applicable.

^a^ Missing data for 2 patients (1 in 2015/16, 1 in 2017/18).

^b^ Missing data for 4 patients (1 in 2015/16, 3 in 2017/18).

^c^ Missing data for 2 patients (1 in 2015/16, 1 in 2016/17).

^d^ Missing data for 3 patients (1 in 2015/16, 2 in 2017/18).

^e^ Missing data for 5 patients (2 in 2015/16, 1 in 2016/17, 2 in 2017/18).

^f^ Missing data for 7 patients (2 in 2015/16, 5 in 2017/18).

^g^ Percentage calculated among cases positive for influenza.

^h^ Percentage calculated among cases positive for influenza A.

^i^ In the 2017/2018 season, one case of double infection was diagnosed, positive for both A/H1 and B.

^j^ Percentage calculated among cases positive for influenza B.

Most patients (92.5%) had at least one underlying condition at the time of hospital admission, the most frequent being cardiovascular disease, present in 78.3% of cases, followed by diabetes (32.5%), obesity (29.2%), and chronic lung disease (22.6%). Other types of comorbidities were less frequent, being reported in less than 15% of patients (Table 1). Patients had a median number of 2 comorbidities, with an overall range of 0‐6 underlying diseases. The median (IQR) number of visits to the general practitioner during the past 12 months was 1 (0, 3), with a range of 0‐10 visits. None of the patients reported an episode of laboratory‐confirmed influenza in the previous season.

**FIGURE 1:**
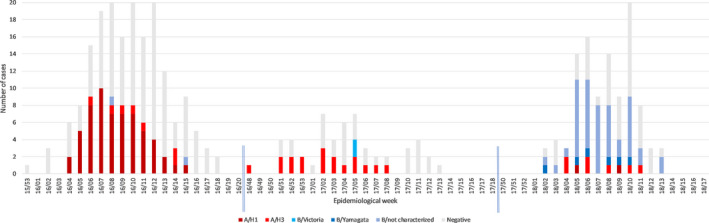
Distribution of influenza cases by virus type and subtype/lineage in elderly patients from Bucharest Romania in the three consecutive studied seasons: 2015/16, 2016/17, and 2017/18

In the season 2015/16, laboratory‐confirmed influenza cases were identified between weeks 04/2016 and 15/2016, in the following season between weeks 48/2016 and 08/2017, and in the third season between weeks 02/2018 and 13/2018. Influenza virus type and subtype distribution by season is presented in Figure 1.

Overall, 149 (42.7%) of the tested patients were positive for influenza. During the first two influenza seasons investigated, we noticed a predominant circulation of influenza A viruses in the study population (97.1% of all influenza‐positive cases in 2015/16 and 90.0% in 2016/17). We saw an apparent switch between seasons from A(H1N1)pdm09 (89.4% of influenza A cases in 2015/16) to A(H3N2) (100% of influenza A cases in 2016/17), while in the third season, there was a distinct predominance of influenza B (86.9% of all influenza‐positive cases in 2017/18).

The full hemagglutinin gene was sequenced from 45 influenza viruses isolated from 2015 to 2018. All characterized A(H1N1)pdm09 viruses (n = 21) fell into clade A/California/7/2009 with the amino acid variations that define group 6B viruses (A/South Africa/3626/2013). The majority of the sequences (13 out of the 15) from the 2017/18 influenza season also possessed the amino acid substitutions of the new emerging subgroups 6B.1 (eg, A/Michigan/45/2015) and 6B.2 subgroup (eg, A/England/377/2015). The 18 HA sequences of A(H3N2) viruses analyzed for amino acid substitutions fell within genetic group 3C, subgroup 3C.2a like A/Hong Kong/5738/2014. The two viruses tested in 2016/17 (B/Victoria lineage) fell into the B/Brisbane/60/2008 genetic clade and all (n = 4) viruses tested in 2017/18 (B/Yamagata lineage) fell in clade 3 (B/Phuket/3073/2013). During the study period, 20 (13.4%) strains of influenza virus were tested for antiviral resistance; all strains showed susceptibility to oseltamivir.

Among the SARI criteria, fever (94.6%), malaise (90.6%), and myalgia (71.7%) were the most frequently encountered signs or symptoms in patients testing positive for influenza, and specifically fever (OR = 3.0) and odynophagia (OR = 1.8) were significantly predictive for testing positive for influenza (Table 2). The distribution of comorbidities was comparable between patients with and without influenza (Table 2). We observed no significant differences in clinical signs or symptoms, or in the distribution of comorbidities between patients with influenza A and influenza B (Table 3).

**TABLE 2:** Distribution of SARI criteria and other patient characteristics in patients testing negative and positive for influenza

Studied variable	Patients testing negative for influenza (n = 200)	Patients testing positive for influenza (n = 149)	Overall (n = 349)	Statistical analysis
SARI criteria				
Fever^a^	169 (85.4%)	141 (94.6%)	310 (89.3)	OR = 3.0, 95% CI: 1.3‐6.6, *P* = .008
Feverishness^b^	11 (5.5%)	14 (9.4%)	25 (7.2%)	*P* = .209
Malaise^b^	186 (93.0%)	135 (90.6%)	321 (92.2%)	*P* = .418
Headache^c^	129 (65.2%)	97 (65.5%)	226 (65.3%)	*P* = 1.000
Myalgia^a^	135 (68.2%)	106 (71.1%)	241 (69.5%)	*P* = .638
Altered clinical state^a^	138 (69.7%)	98 (65.8%)	236 (68.0%)	*P* = .486
Cough^b^	190 (95.5%)	143 (96.0%)	333 (95.7%)	*P* = 1.000
Odynophagia^a^	64 (32.3%)	68 (45.6%)	132 (38.0%)	OR = 1.8, 95% CI: 1.1‐2.7, *P* = .014
Dyspnea^c^	129 (65.5%)	103 (69.1%)	232 (67.1%)	*P* = .491
Smoking status^b^				
Never smoked	139 (69.8%)	109 (73.2%)	248 (71.3%)	*P* = .767
Former smoker	45 (22.6%)	29 (19.5%)	74 (21.3%)	
Current smoker	15 (7.5%)	11 (7.4%)	26 (7.5%)	
Vaccinated against influenza in the current season	15 (7.5%, 95% CI: 4.3%‐12.1%)	7 (4.7%, 95% CI: 1.9%‐9.5%)	22 (6.3%, 95% CI: 4.0%‐9.4%)	*P* = .375
Comorbidities				
Patients with comorbidities^a^	184 (92.9%)	137 (91.9%)	321 (92.5%)	*P* = .837
Number of comorbidities, median (range)^a^	2 (0‐6)	2 (0‐4)	2 (0‐6)	*P* = .133
Frailty index, median (IQR)^a^	0.2 (0.1, 0.3)	0.2 (0.1, 0.3)	0.2 (0.1, 0.3)	N/A
Cardiovascular disease^d^	155 (78.7%)	115 (77.7%)	270 (78.3%)	*P* = .895
Diabetes^d^	59 (29.9%)	53 (35.8%)	112 (32.5%)	*P* = .296
Obesity^a^	57 (28.8%)	41 (27.5%)	98 (28.2%)	*P* = .811
Chronic pulmonary disease^d^	52 (26.4%)	26 (17.6%)	78 (22.6%)	*P* = .068
Chronic liver disease^c^	14 (7.1%)	7 (4.7%)	21 (6.1%)	*P* = .496
Chronic kidney disease^e^	36 (18.4%)	11 (7.4%)	47 (13.7%)	OR = 0.4, 95% CI: 0.2‐0.7, *P* = .004
Hematologic cancer^a^	4 (2.0%)	5 (3.4%)	9 (2.6%)	*P* = .506
Non‐hematologic cancer^a^	14 (7.1%)	17 (11.4%)	31 (8.9%)	*P* = .185
Rheumatologic disease^f^	37 (19.0%)	11 (7.5%)	48 (14.0%)	OR = 0.4, 95% CI: 0.2‐0.7, *P* = .003
Immune suppression^a^	8 (4.0%)	1 (0.7%)	9 (2.6%)	*P* = .084
Antiviral treatment	108 (54.0%)	121 (81.2%)	229 (65.6%)	*P* < .001
Death during hospitalization	4 (2.0%, 95% CI: 0.5%‐5.0%)	4 (2.7%, 95% CI: 0.7%‐6.7%)	8 (2.3%, 95% CI: 1.0%‐4.5%)	*P* = .728

Note

All data are presented as number (percentage), unless otherwise specified. Statistical analysis was performed with the two‐tailed chi‐squared test or Mann‐Whitney's *U* test. Frailty index was calculated by dividing the number of active comorbidities by the total number of comorbidities assessed for each patient.

Abbreviations: 95% CI, 95% confidence interval; N/A, not applicable; OR, odds ratio; SARI, severe acute respiratory infection.

^a^ Missing data for 2 patients.

^b^ Missing data for 1 patient.

^c^ Missing data for 3 patients.

^d^ Missing data for 4 patients.

^e^ Missing data for 5 patients.

^f^ Missing data for 7 patients.

**TABLE 3:** Distribution of SARI criteria and other patient characteristics in patients with laboratory‐confirmed influenza A and B

Studied variable	Patients positive for influenza A (n = 93)	Patients positive for influenza B (n = 56)	Overall (n = 149)	Statistical analysis
Fever	87 (93.5%)	54 (96.4%)	141 (94.6%)	*P* = .711
Feverishness	3 (3.2%)	11 (19.6%)	14 (9.4%)	OR = 7.3, 95% CI: 2.0‐27.6, *P* = .002
Malaise	84 (90.3%)	51 (91.1%)	135 (90.6%)	*P* = 1.000
Headache^a^	64 (69.6%)	33 (58.9%)	97 (65.5%)	*P* = .214
Myalgia	68 (73.1%)	38 (67.9%)	106 (71.1%)	*P* = .576
Altered clinical state	63 (67.7%)	35 (62.5%)	98 (65.8%)	*P* = .593
Cough	89 (95.7%)	54 (96.4%)	143 (96.0%)	*P* = 1.000
Odynophagia	41 (44.1%)	27 (48.2%)	68 (45.6%)	*P* = .734
Dyspnea	65 (69.9%)	38 (67.9%)	103 (69.1%)	*P* = .855
Smoking status				
Never smoked	73 (78.5%)	36 (64.3%)	109 (73.2%)	*P* = .091
Former smoker	13 (14.0%)	16 (28.6%)	29 (19.5%)	
Current smoker	7 (7.5%)	4 (7.1%)	11 (7.4%)	
Vaccinated against influenza in the current season	6 (6.5%, 95% CI: 2.4%‐13.7%)	1 (1.8%, 95% CI: 0.0%‐9.6%)	7 (4.7%, 95% CI: 1.9%‐9.5%)	*P* = .254
Comorbidities				
Patients with comorbidities^b^	86 (92.5%)	51 (91.1%)	137 (91.9%)	*P* = .764
Number of comorbidities, median (range)^b^	2 (0‐4)	2 (0‐4)	2 (0‐4)	N/A
Frailty index, median (IQR)^b^	0.2 (0.1, 0.3)	0.2 (0.1, 0.3)	0.2 (0.1, 0.3)	N/A
Cardiovascular disease^c^	73 (78.5%)	42 (76.4%)	115 (77.7%)	*P* = .839
Diabetes^c^	33 (35.9%)	20 (35.7%)	53 (35.8%)	*P* = .999
Obesity^b^	23 (24.7%)	18 (32.1%)	41 (27.5%)	*P* = .348
Chronic pulmonary disease^c^	15 (16.3%)	11 (19.6%)	26 (17.6%)	*P* = .659
Chronic liver disease^d^	2 (2.2%)	5 (8.9%)	7 (4.7%)	*P* = .105
Chronic kidney disease^e^	5 (5.4%)	6 (10.7%)	11 (7.4%)	*P* = .333
Hematologic cancer^b^	4 (4.3%)	1 (1.8%)	5 (3.4%)	*P* = .651
Non‐hematologic cancer^b^	11 (11.8%)	6 (10.7%)	17 (11.4%)	*P* = .999
Rheumatologic disease^f^	9 (9.8%)	2 (3.6%)	11 (7.5%)	*P* = .211
Immune suppression^b^	0 (0.0%)	1 (1.8%)	1 (0.7%)	N/A
Antiviral treatment	78 (83.9%)	43 (76.8%)	121 (81.2%)	*P* = .289
Death during hospitalization^g^	4 (4.3%, 95% CI: 1.2%‐10.6%)	0 (0.0%, 95% CI: 0.0%‐6.4%)	4 (2.7%, 95% CI: 0.7%‐6.7%)	*P* = .297

Note

All data is presented as number (percentage), unless otherwise specified. Statistical analysis was performed with the two‐tailed chi‐squared test or Mann‐Whitney's *U* test. Frailty index was calculated by dividing the number of active comorbidities by the total number of comorbidities assessed for each patient.

Abbreviations: 95% CI, 95% confidence interval; N/A, not applicable; OR, odds ratio; SARI, severe acute respiratory infection.

^a^ Missing data for 1 patient.

^b^ Missing data for 2 patients.

^c^ Missing data for 4 patients.

^d^ Missing data for 3 patients.

^e^ Missing data for 5 patients.

^f^ Missing data for 7 patients.

^g^ Death occurred in 3 patients with influenza A/H1 and one with A/H3, all in the 2015/16 season. Four more deaths occurred in patients who tested negative for influenza in the 2017/18 season.

Overall, 65.6% of the patients included in the study received antiviral treatment with oseltamivir during hospital admission, and the proportion was higher in patients with laboratory‐confirmed influenza (81.2%). The clinical course of disease was generally favorable. However, eight patients died during hospital admission, all having at least one underlying condition; of them, four had been confirmed with influenza, and four were negative for influenza. All four deaths that occurred in patients with laboratory‐confirmed influenza were caused by influenza A [three A(H1N1)pdm09 and one A(H3N2)]; none of these four patients had been vaccinated against influenza, three were females, all were aged 76 years old, and had a range of 1‐3 comorbidities, as follows: three had diabetes, two had chronic lung disease, one had cardiovascular disease and one had cancer. The overall calculated case fatality in this study was 2.3%: 2.7% in patients with laboratory‐confirmed influenza and 2.0% in the patients testing negative (Table 2). Case fatality was higher in the 2015/16 influenza season than in the subsequent seasons (3.7%, 0.0%, 1.0%) (Table 1).

A total of 22 (6.3%) patients had been vaccinated against influenza in the respective current season, all with standard‐dose inactivated trivalent vaccine, as follows: 20 with Influvac (Abbott Biologicals BV) and 2 with Vaxigrip (Sanofi Pasteur SA). Among these, 15 tested negative for influenza. Out of the remaining seven that tested positive, six were diagnosed in the first season (2015/16) with influenza A [4 A(H1N1)pdm09 and 2 A(H3N2)], and one in the third season (2017/18) with influenza B (no lineage data available). They had been vaccinated within a range of 6 to 24 weeks prior to symptom onset. Three were male and four were female, and their ages ranged from 66 to 81 years old. Only one of them had no comorbidities, the others presenting 0‐3 underlying conditions, as follows: five had cardiovascular disease, two had diabetes mellitus, two had cancer, one had chronic kidney disease, one had rheumatologic disease, and one was obese; no deaths were recorded among these seven vaccine failures during their hospitalization for influenza.

## 4 DISCUSSION

In this paper, we have reported the clinical and epidemiological characteristics of elderly patients admitted in our hospital for SARI in three consecutive influenza seasons from Bucharest, Romania.

Our study has highlighted a high burden of comorbidities in elderly patients presenting with SARI during winter season in Romania, over 90% with at least one comorbid disease. The synergy between old age, potential immune hyporeactivity, and the presence of multiple chronic diseases may put this group of patients at an increased risk of decompensating the underlying condition during influenza infection, particularly cardiovascular diseases,^9^ which were highly prevalent in our study population, or of developing bacterial pneumonia.^10^, ^11^, ^12^, ^13^

Clinical characteristics of influenza did not differ significantly between elderly patients with influenza A and B in our study. This is in line with previous research from our institute in children^14^ and from France in all age groups, showing that clinical characteristics are mostly indistinguishable between viral types and subtypes.^15^ Among the SARI criteria, fever and odynophagia associated higher odds of testing positive for influenza in our study; this is consistent with the data reported by Falsey et al, which showed that the combination of fever with cough or odynophagia exhibited the best balance of sensitivity and specificity for diagnosing influenza.^16^ Falsey et al also suggested that in elderly patients, the threshold for defining fever could be as low as 37.3°C.^16^

Overall, influenza A(H1N1)pdm09 was the main viral subtype circulating in 2015/16, followed by A(H3N2) in 2016/17 and B (supposedly mainly B/Yamagata) in 2017/18 in our study. These data are in line with that reported on a national level by the Romanian National Center for Surveillance and Control of Transmissible Diseases, with certain particularities, discussed below.

For the 2015/16 season, influenza A was the main circulating type in Romania in all age groups, accounting for 90.6% of all influenza cases reported and analyzed on a national level,^17^ compared to 97.1% in our study of elderly patients, and the main subtype was A(H1N1)pdm09, accounting for 93.5% of all subtyped A strains nationally,^17^ compared with 89.4% in our study. The A(H3N2) subtype was relatively less frequently identified at the national level (6.5%^17^ compared with 10.6% in our study), while the circulation of B strains, reported at 9.4% in the country,^17^ was almost negligible in our study (2.9%) for the 2015/16 season. These slight differences might arise from a different sampling frame (one tertiary care hospital analyzing hospitalized SARI in our study, compared to an ILI‐based national surveillance system^17^ in the country as a whole), but they might also reflect to a certain degree a particularity of the patients admitted in our hospital that is a referral hospital for the south of Romania.^18^

For the 2016/17 season, the data from our current study confirmed to some extent our previous report regarding the exclusive co‐circulation of A(H3N2) and B/Victoria.^19^ However, in our study of elderly patients the season was dominated by the circulation of A viruses (90.0% compared with the national estimate of 65.7% in all age groups^20^ and compared to our previous report of 33.9% in all age groups with an emphasis on children^19^), suggesting that A viruses predominated in elderly patients in 2016/17; A(H3N2) accounted for 100% of all circulating A viruses in 2016/17 in the current study and in our previous report.^19^ Data from Bulgaria and Poland also report the predominance of influenza A virus (97.5%^21^ and 95.5%^22^ of all influenza cases, respectively), and specifically the A(H3N2) subtype in the 2016/17 season, but A(H1N1)pdm09 was also present in these two countries, to a lower extent.^21^, ^22^ Our data are also in agreement with a recent meta‐regression analysis reporting that type B influenza viruses are somewhat less frequent in elderly patients compared to the other age groups.^23^

For the 2017/18 season, the predominance of B viruses was evident in our SARI study, accounting for 86.9% of all influenza cases, but the rate was higher than the one reported for ILI‐based surveillance at the national level (66.5%).^24^ In our study, lineage determination was only performed for a small proportion of B viruses circulating during this season (11.3%, 6/53 cases), but based on the results available from a different study concomitantly conducted in our hospital in the 2017/18 season, B/Yamagata appeared to be the main circulating strain in patients of all ages.^25^

Case fatality was overall low, being higher in 2015/16 (3.7%), and lower in the following seasons, 0% in 2016/17 and 1.0% in 2017/18, and there were no significant changes in the healthcare system to account for this difference. Out of the four deaths recorded in patients with laboratory‐confirmed influenza, all occurred during the 2015/16 season, 3 occurred in patients with A(H1N1)pdm09 and one with A(H3N2) infection. The Romanian National Center for Surveillance and Control of Transmissible Diseases has reported that for patients with SARI in the 2015/16 influenza season, testing positive for A(H1N1)pdm09 associated a fourfold increased risk of death, compared to infection due to other viral subtypes, and that the overall severity of influenza was high that season.^26^ Furthermore, based on the same national report, the general fatality rate for SARI in 2015/16 was reported at 20.7% and at 49.0% for SARI confirmed as influenza,^26^ and another study has reported a case fatality proportion of 39.8% (95% CI: 29.5%‐50.8%) for that same season for SARI confirmed as influenza.^27^ Differences in the lower case fatality reported in our study compared to the national estimate could potentially be due to selection testing bias of severe cases in the conventional surveillance system, leading to an artificial increase in the reported fatality ratio. These differences may also be partly explained by the specific profile of our institute, which is a major reference center for infectious diseases in Romania, and is well equipped to manage influenza cases in a timely manner, and to institute specific antiviral treatment promptly after hospital admission of SARI cases. In our study, 65.6% of patients admitted with SARI received antiviral treatment. In SARI cases confirmed as influenza, the percentage was higher, 81.2%, whereas in patients who tested negative for influenza, 54.0% received an antiviral, but treatment was stopped after influenza was ruled out and an alternative diagnosis was established.

**FIGURE 2:**
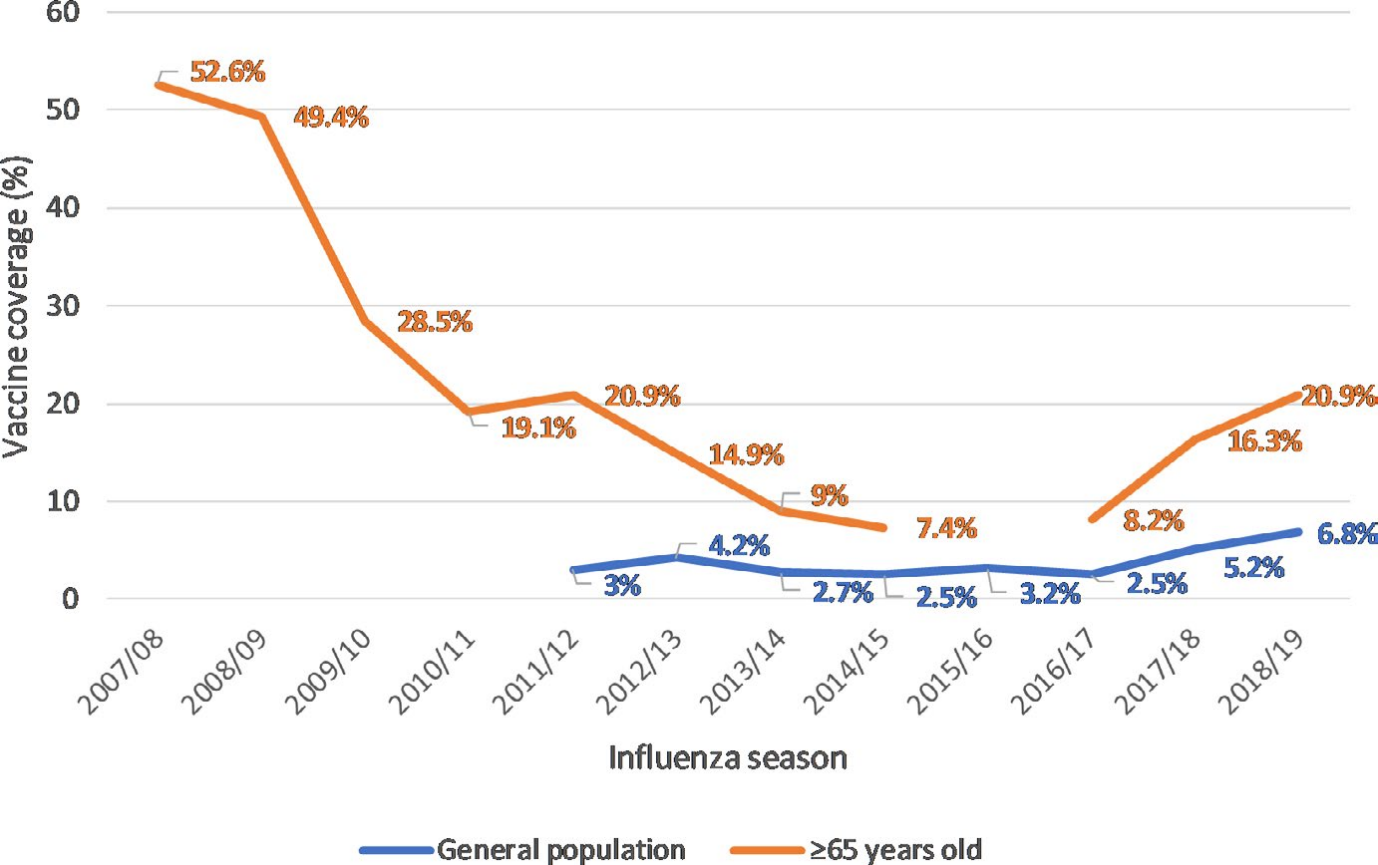
Influenza vaccine coverage in Romania in the general population and in elderly patients, 2007/08 to 2018/19

In Romania, influenza vaccination is provided each year by health authorities, through the general practitioners, to priority risk groups as defined by the World Health Organization (WHO). However, vaccine coverage remains generally low for most risk groups, and for elderly patients in particular, with a marked decrease in the immediate post‐2009‐pandemic period (49.4% to 19.1% for elderly patients in 2008/09 vs 2010/11), as also reported for other countries,^28^ and a slowly increasing trend in the past 3 years (Figure 2).^28^, ^29^, ^30^, ^31^, ^32^ In our study population, the influenza vaccination uptake was low over the three seasons. In the 2015/16 season, the vaccination uptake was 7.9% (95% CI: 4.5%‐12.7%) in our study population, and the national reported rate for elderly patients was 10.3%.^17^ For the 2016/17 season, the influenza vaccine coverage in our study was 3.8% (95% CI: 0.5%‐13.0%), and the national reported coverage was 8.2% in elderly patients.^20^ For the 2017/18 season, the vaccine coverage in our study (4.8%, 95% CI: 1.6%‐10.8%) was lower that the coverage in elderly patients on a national level, reported at 16.3%.^24^ Given the overall low number of vaccinated patients included in our study, we cannot conclude whether there was a protective effect of vaccination against hospital admission for SARI. Stronger data from Australia have shown that an influenza vaccine coverage of 80.2% in elderly patients was able to prevent 49.5% of hospital admissions due to influenza in the 2015 influenza season.^33^

We recorded a total number of seven cases of influenza in patients who had been vaccinated in the respective current season; most of these vaccine failures occurred in the 2015/16 season. The adjusted influenza vaccine effectiveness (IVE) in elderly patients (defined as 60 years and older) for the 2015/16 season in Spain was low (20.2% for preventing hospital admission with A(H1N1)pdm09),^34^ similar to the data from the I‐MOVE+ study in Poland, reporting an overall vaccine effectiveness of 21.0%.^35^ For Romania, the low vaccine uptake for the previous influenza seasons did not allow an adequate calculation of the IVE, with very small numbers of cases and controls included in the formula, which led to an adjusted IVE against hospitalized A(H1N1)pdm09 infection reported at −22.6%, but with a very wide confidence interval (−490.3% to 74.6%).^36^

Published studies have shown that standard‐dose influenza vaccines may induce lower hemagglutination‐inhibition HAI titers and seroprotection rates in elderly patients.^37^, ^38^ In Romania, high‐dose influenza vaccines have so far not been available and therefore, the data we are reporting here refer to elderly patients who had received standard‐dose inactivated trivalent vaccines.

Our study's main strength is that it employed systematic screening of all elderly patients admitted for SARI to one tertiary care hospital in Romania, applying the same standardized methodology throughout three consecutive influenza seasons, thus allowing an analysis of the differences from season to season in terms of clinical characteristics, viral type/subtype circulation, case fatality, and use of healthcare resources.

This study also had a number of limitations. The series is relatively small, 349 patients from one single tertiary care center over the course of three consecutive influenza seasons. The overall low vaccination uptake in our study population and in the country precluded us from performing an analysis of vaccine effectiveness for this study site. Also, lineage determination was only performed for a small proportion of B viruses circulating in the third influenza season investigated, which did not allow an exact quantification of the circulation of B/Victoria vs. B/Yamagata strains, but our data, corroborated with other information from field literature, showed that in the 2017/18 season in the Bucharest‐Ilfov region B/Yamagata accounted for 90.4% of all characterized B strains circulating in hospitalized influenza.^25^

Continued surveillance of influenza is needed in order to inform the best local practices. In elderly patients from our setting, given the high burden of comorbidities that we have characterized, interdisciplinary management and good control of underlying diseases are important, and should be coupled with targeted interventions to substantially increase vaccine uptake.

## 5 CONCLUSIONS

Among elderly patients admitted to the hospital with SARI in Bucharest Romania, influenza A(H1N1)pdm09 was the main viral subtype circulating in 2015/16, A(H3N2) in 2016/17, and B influenza in 2017/18, respectively. Case fatality was low in general, but it was highest during the 2015/16 season with predominant circulation of influenza A(H1N1)pdm09. Vaccination uptake in elderly patients from our study population was low, highlighting the importance of promoting vaccination in the elderly patients in general and in those with underlying conditions that present higher risk for severe disease.

## CONFLICTS OF INTEREST

DP is the technical project manager for the GIHSN project funded by Sanofi Pasteur and Foundation for Influenza Epidemiology, principal investigator of the I‐MOVE+ study funded through the European Union's HORIZON 2020 research and innovation program, and technical project manager for the DRIVE study, that has received support from the EU/EFPIA Innovative Medicines Initiative 2 Joint Undertaking (DRIVE, grant n° 777363). No conflict of interest.

AnSC is the principal investigator of the I‐MOVE+ study 2017/18 funded through the European Union's HORIZON 2020 research and innovation program, member of the research team of the DRIVE study, that has received support from the EU/EFPIA Innovative Medicines Initiative 2 Joint Undertaking (DRIVE, grant n° 777363), and subinvestigator in influenza clinical trials by Shionogi and Roche; no conflict of interest.

AEI is the coordinator of the molecular detection of influenza type and subtype by real‐time reverse transcription PCR through the IMOVE+ project. No conflict of interest.

ML is the coordinator of the genetic characterization of influenza strains. No conflict of interest.

CMC is the coordinator of the isolation of influenza viruses in cell culture and antigenic characterization in the IMOVE+ study. No conflict of interest.

MEM is the coordinator of the antiviral resistance in IMOVE+ study. No conflict of interest.

MN is the principal investigator of the I‐MOVE+ study 2016/18 funded through the European Union's HORIZON 2020 research and innovation program.

VA is the member of the research team of the GIHSN project funded by Sanofi Pasteur and Foundation for Influenza Epidemiology, member of the research team of the DRIVE study, that has received support from the EU/EFPIA Innovative Medicines Initiative 2 Joint Undertaking (DRIVE, grant n° 777363), and reports lectures and advisory boards for Sanofi and Astra‐Zeneca, outside of the submitted work. No conflict of interest.

MDC No conflict of interest.

ASC is the member of the research team of the GIHSN project funded by Sanofi Pasteur and Foundation for Influenza Epidemiology, member of the research team of the DRIVE study, that has received support from the EU/EFPIA Innovative Medicines Initiative 2 Joint Undertaking (DRIVE, grant n° 777363), principal investigator in influenza clinical trials by Shionogi and Roche, and reports lectures for Sanofi, outside of the submitted work. No conflict of interest.

OS is the member of the research team of the GIHSN project funded by Sanofi Pasteur and Foundation for Influenza Epidemiology, principal investigator for adults in the DRIVE study, that has received support from the EU/EFPIA Innovative Medicines Initiative 2 Joint Undertaking (DRIVE, grant n° 777363), subinvestigator in influenza clinical trials by Shionogi and Roche, and reports lectures for Sanofi, outside of the submitted work. No conflict of interest.

## AUTHOR CONTRIBUTIONS

All authors had equal contributions.
